# Supplementary material for: The detection of trans gene fragments of hEPO in gene doping model mice by Taqman qPCR assay
Source: PeerJ. 2020 Feb 25;8:e8595. doi: 10.7717/peerj.8595 (PMC7047860; doi:10.7717/peerj.8595)

# Blood

IV:1h

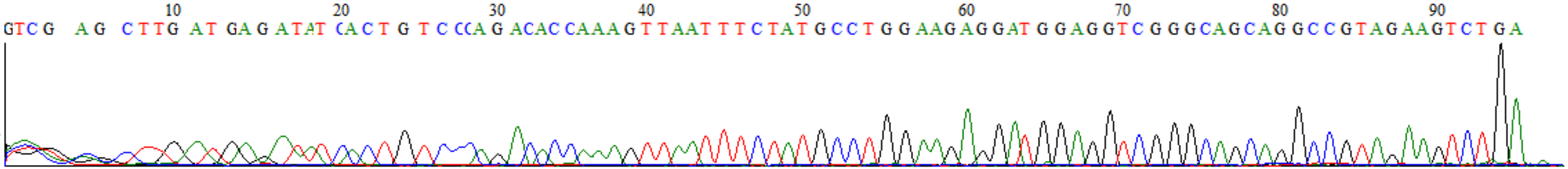

IV:2h

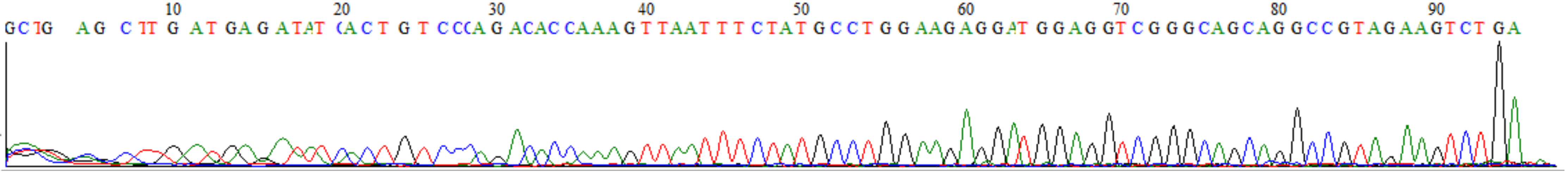

IV:3h

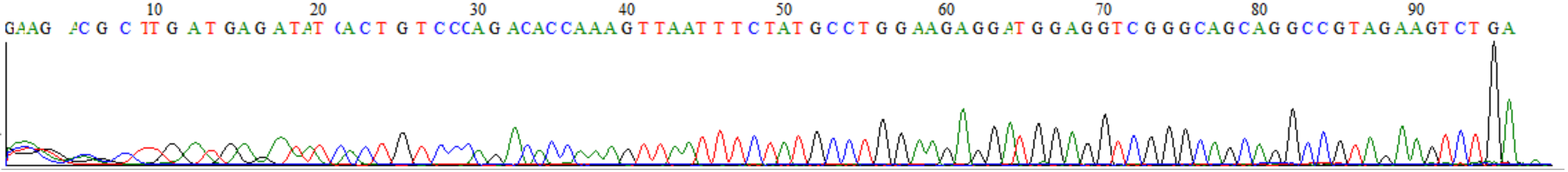

IM:1h

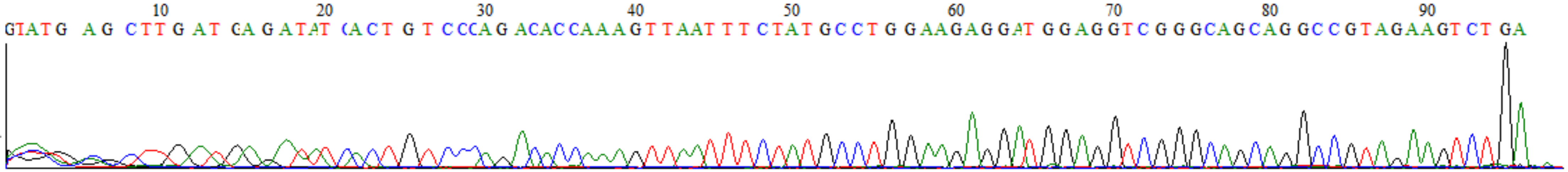

IM:2h

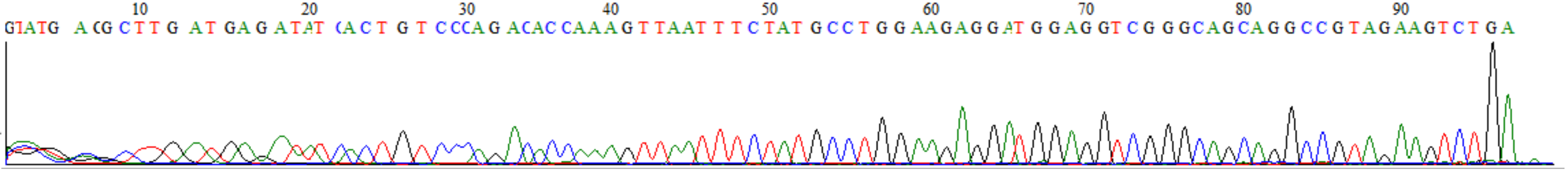

IM:3h

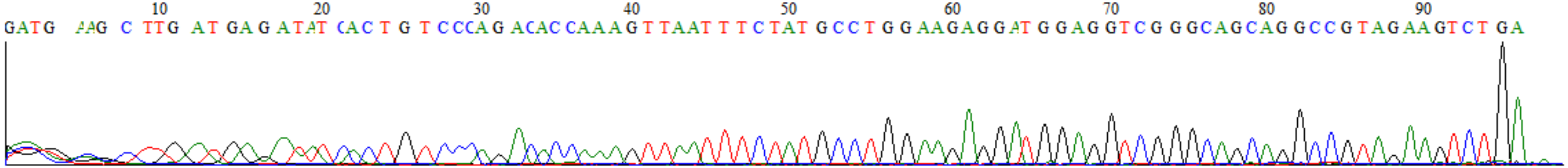

IP:1h

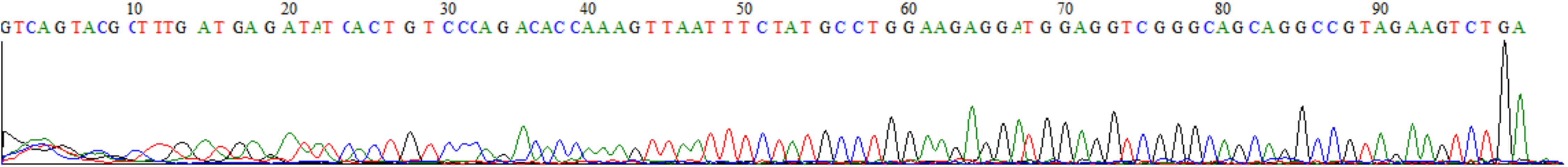


IP:2h

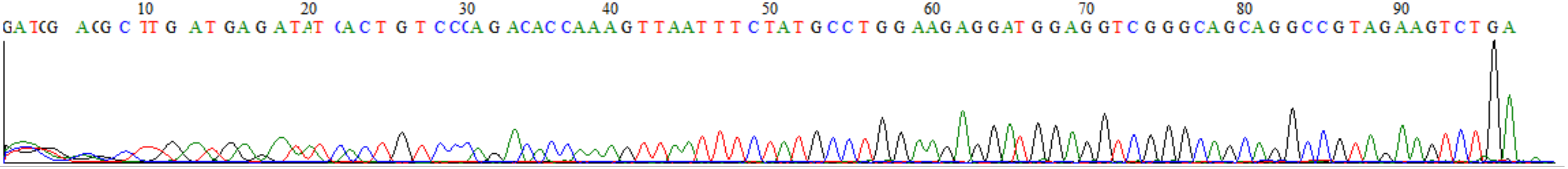


IP:3h

# Stool

IV:1h

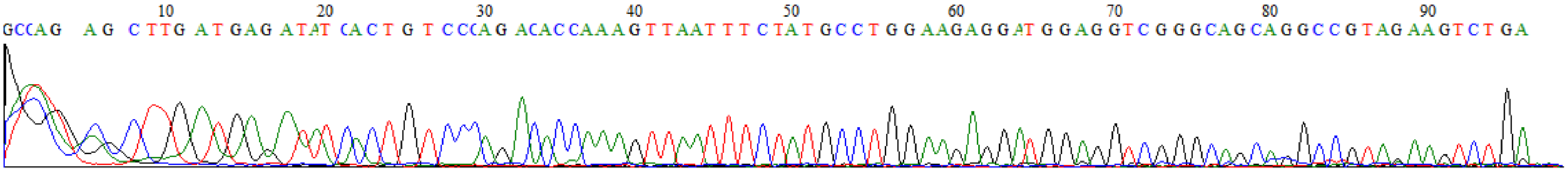

IV:2h

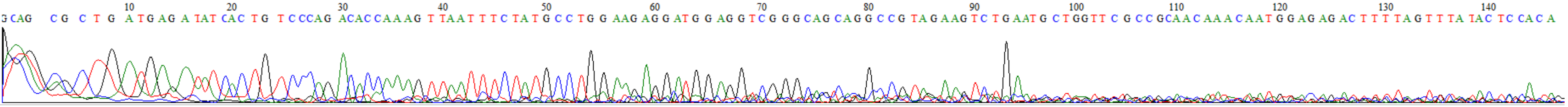

IV:3h

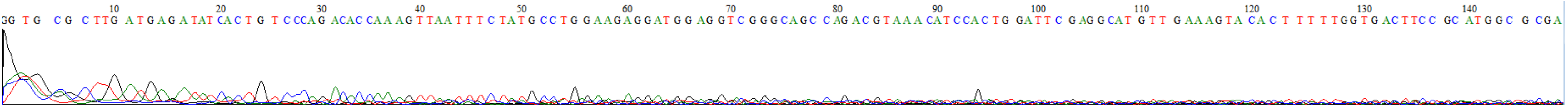

IM:1h

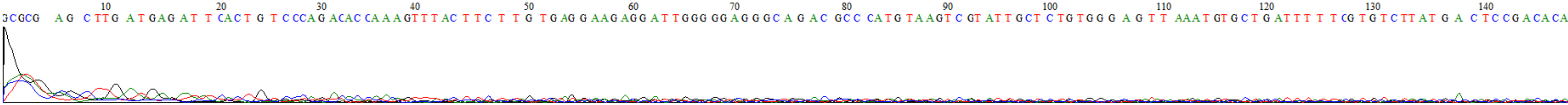

IM:2h

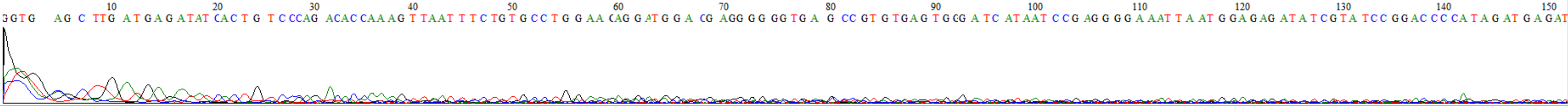

IM:3h

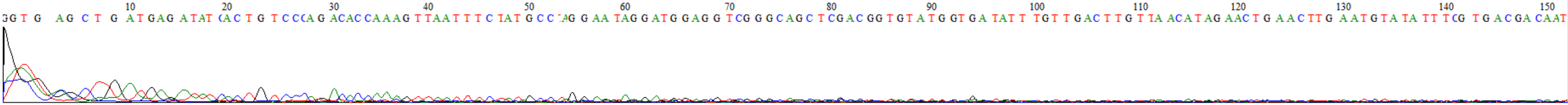

IP:1h

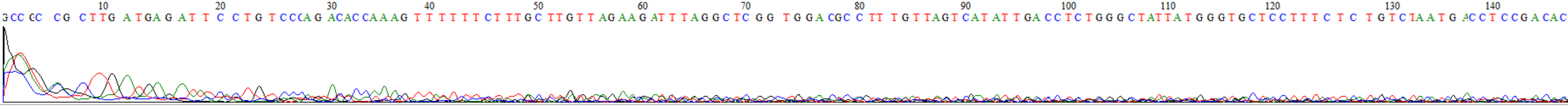

IP:2h

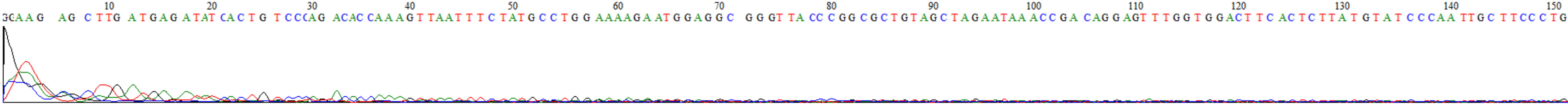

IP:3h

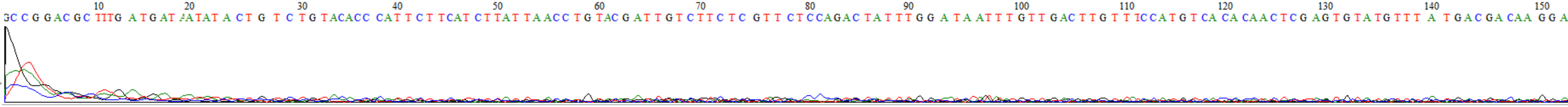

# Blood

IV:1h

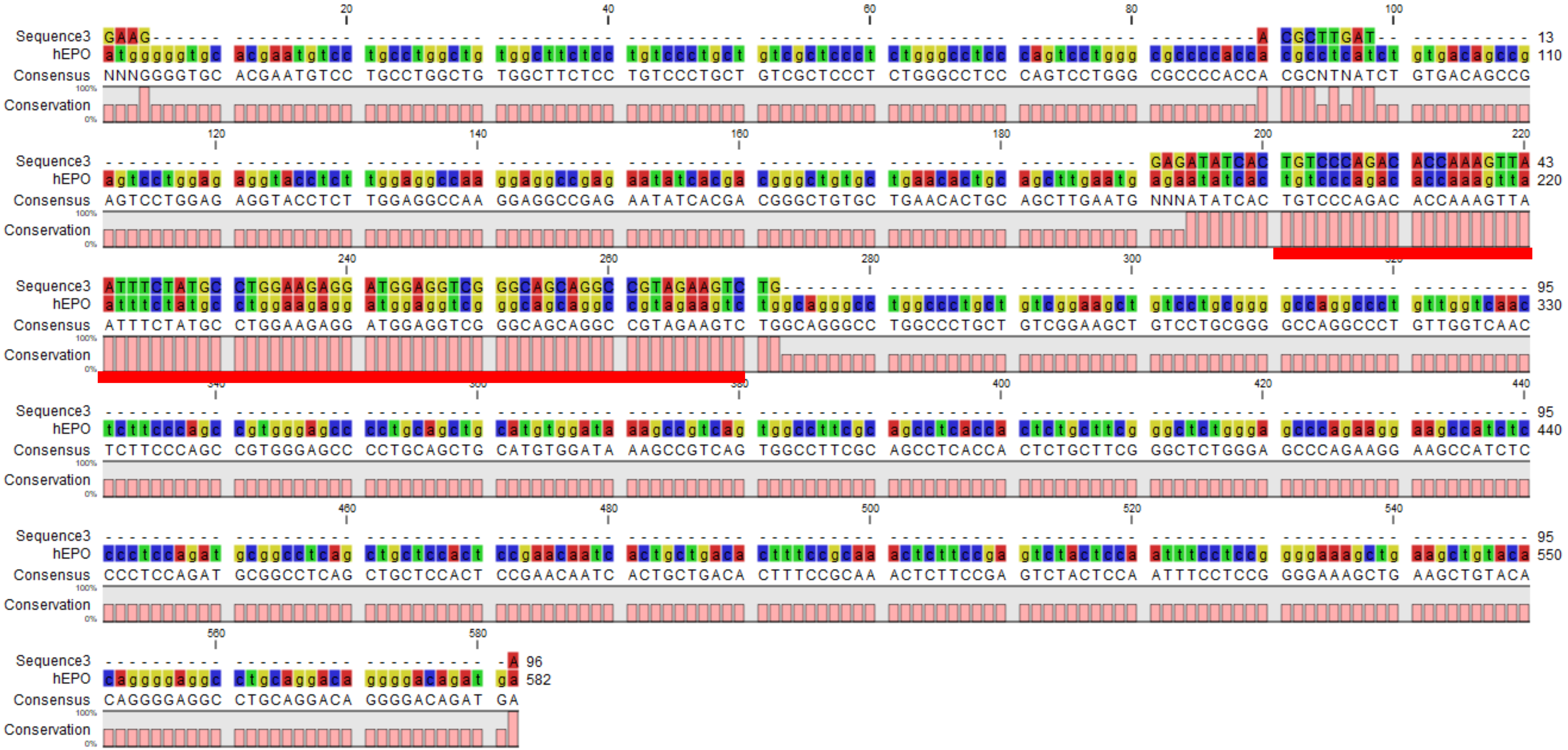

# IV:2h

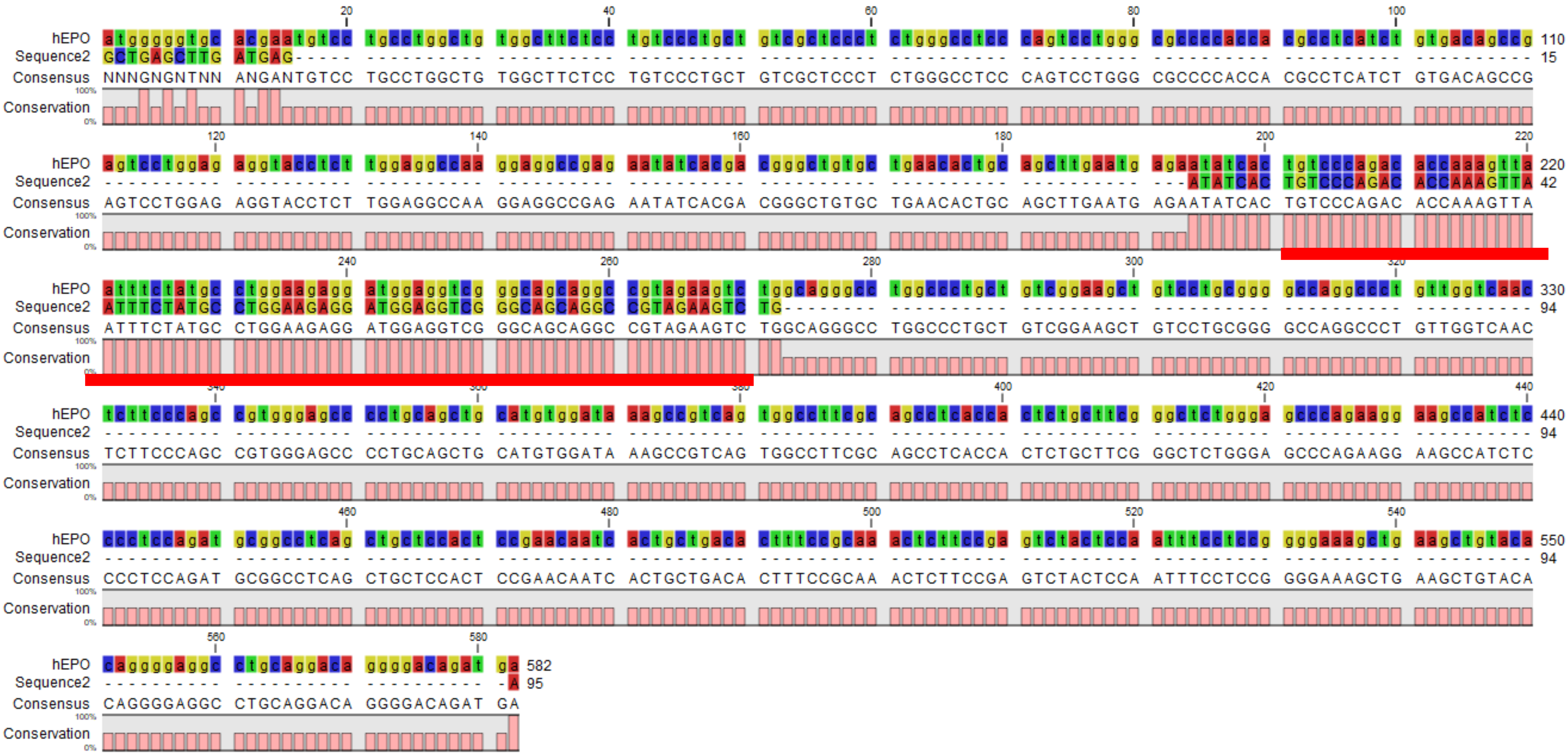

20 40 60 80 100
hEPO atgggggtgc acgaatgtcc tgcctggctg tggcttctcc tgtccctgct gtcgctccct ctgggcctcc cagtcctggg cgccccacca cgcctcatct gtgacagccg 110
Sequence2 GCTGAGCTTG ATGAG----- 15
Consensus NNNGNGNTNN ANGANTGTCC TGCCTGGCTG TGGCTTCTCC TGTCCCTGCT GTCGCTCCCT CTGGGCCTCC CAGTCCTGGG CGCCCCACCA CGCCTCATCT GTGACAGCCG
Conservation
120 140 160 180 200 220
hEPO agtcctggag aggtacctct tggaggccaa ggaggccgag aatatcacga cgggctgtgc tgaacactgc agcttgaatg agaatatcac tgtcccagac accaaagtta 220
Sequence2 ---ATATCAC TGTCCCAGAC ACCAAAGTTA 42
Consensus AGTCCTGGAG AGGTACCTCT TGGAGGCCAA GGAGGCCGAG AATATCACGA CGGGCTGTGC TGAACACTGC AGCTTGAATG AGAATATCAC TGTCCCAGAC ACCAAAGTTA
Conservation
240 260 280 300 320
hEPO atttctatgc ctggaagagg atggaggtcg ggcagcaggc cgtagaagtc tggcagggcc tggccctgct gtcggaagct gtcctgcggg gccaggccct gttggtcaac 330
Sequence2 ATTTCTATGC CTGGAAGAGG ATGGAGGTCG GGCAGCAGGC CGTAGAAGTC TG-------- 94
Consensus ATTTCTATGC CTGGAAGAGG ATGGAGGTCG GGCAGCAGGC CGTAGAAGTC TGGCAGGGCC TGGCCCTGCT GTCGGAAGCT GTCCTGCGGG GCCAGGCCCT GTTGGTCAAC
Conservation
340 360 380 400 420 440
hEPO tcttcccagc cgtgggagcc cctgcagctg catgtggata aagccgtcag tggccttcgc agcctcacca ctctgcttcg ggctctggga gcccagaagg aagccatctc 440
Sequence2 94
Consensus TCTTCCCAGC CGTGGGAGCC CCTGCAGCTG CATGTGGATA AAGCCGTCAG TGGCCTTCGC AGCCTCACCA CTCTGCTTCG GGCTCTGGGA GCCCAGAAGG AAGCCATCTC
Conservation
460 480 500 520 540
hEPO ccctccagat gcggcctcag ctgctccact ccgaacaatc actgctgaca ctttccgcaa actcttccga gtctactcca atttcctccg gggaaagctg aagctgtaca 550
Sequence2 94
Consensus CCCTCCAGAT GCGGCCTCAG CTGCTCCACT CCGAACAATC ACTGCTGACA CTTTCCGCAA ACTCTTCCGA GTCTACTCCA ATTTCCTCCG GGGAAAGCTG AAGCTGTACA
Conservation
560 580
hEPO caggggaggc ctgcaggaca ggggacagat ga 582
Sequence2 -A 95
Consensus CAGGGGAGGC CTGCAGGACA GGGGACAGAT GA
Conservation

IV:3h

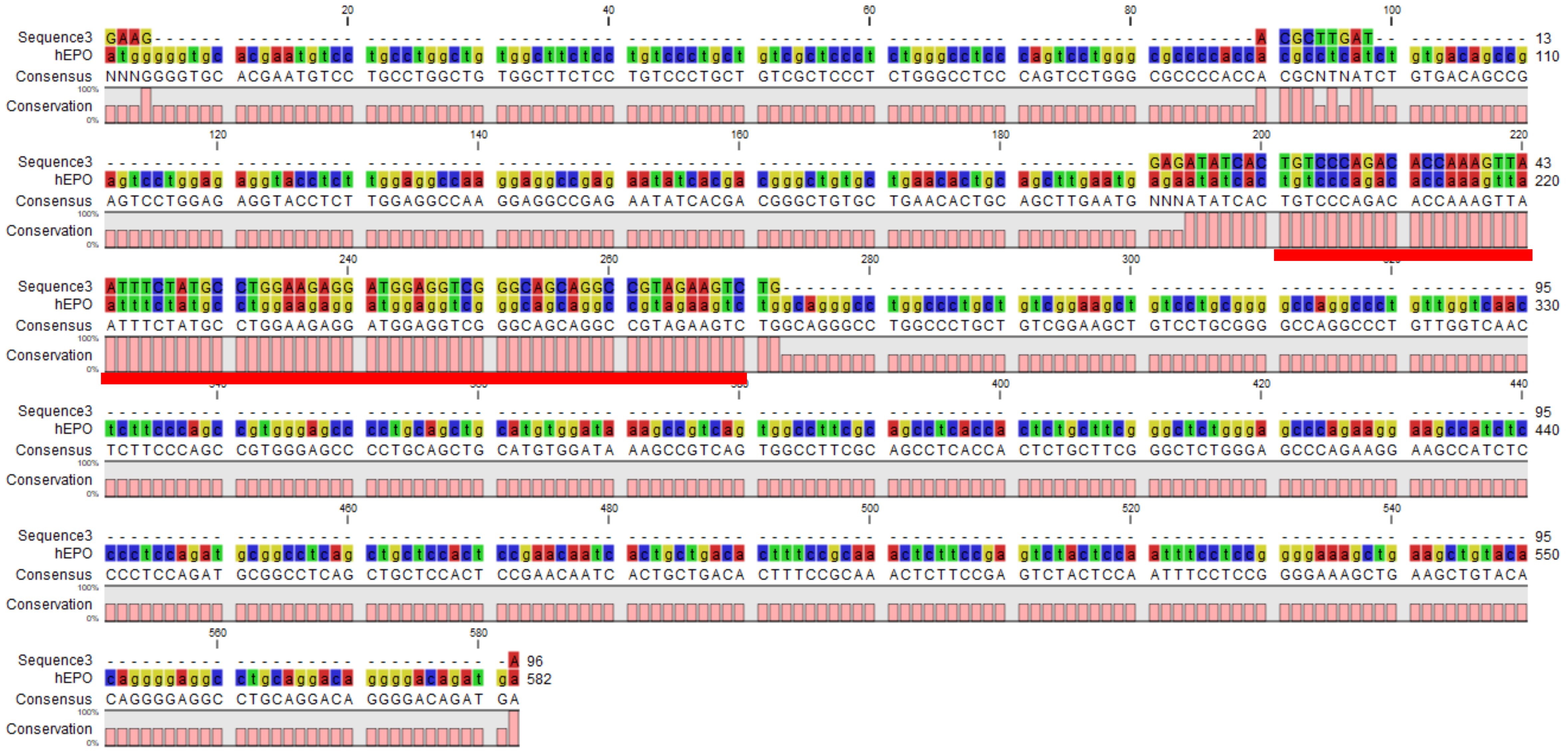
Sequence3 GAAG------ ---------- ---------- ---------- ---------- ---------- ---------- ---------- ---------A CGCTTGAT-- ---------- 13
hEPO atggggtgc acgaatgtcc tgcctggctg tggcttctcc tgtccctgct gtcgctccct ctgggcctcc cagtcctggg cgccccacca cgcctcatct gtgacagccg 110
Consensus NNNGGGTGC ACGAATGTCC TGCCTGGCTG TGGCTTCTCC TGTCCCTGCT GTCGCTCCCT CTGGGCCTCC CAGTCCTGGG CGCCCCACCA CGCNTNATCT GTGACAGCCG
Conservation
Sequence3 ---------- ---------- ---------- ---------- ---------- ---------- ---------- ---------- GAGATATCAC TGTCCCAGAC ACCAAAGTTA 43
hEPO agtcctggag aggtacctct tggaggccaa ggaggccgag aatatcacga cgggctgtgc tgaacactgc agcttgaatg agaatatcac tgtcccagac accaaagtta 220
Consensus AGTCCTGGAG AGGTACCTCT TGGAGGCCAA GGAGGCCGAG AATATCACGA CGGGCTGTGC TGAACACTGC AGCTTGAATG NNNATATCAC TGTCCCAGAC ACCAAAGTTA
Conservation
Sequence3 ATTTCTATGC CTGGAAGAGG ATGGAGGTCG GGCAGCAGGC CGTAGAAGTC TG-------- ---------- ---------- ---------- ---------- ---------- 95
hEPO atttctatgc ctggaagagg atggaggtcg ggcagcaggc cgtagaagtc tggcagggcc tggccctgct gtcggaagct gtcctgcggg gccaggccct gttggtcaac 330
Consensus ATTTCTATGC CTGGAAGAGG ATGGAGGTCG GGCAGCAGGC CGTAGAAGTC TGGCAGGGCC TGGCCCTGCT GTCGGAAGCT GTCCTGCGGG GCCAGGCCCT GTTGGTCAAC
Conservation
Sequence3 ---------- ---------- ---------- ---------- ---------- ---------- ---------- ---------- ---------- ---------- ---------- 95
hEPO tcttcccagc cgtgggagcc cctgcagctg catgtggata aagccgtcag tggccttcgc agcctcacca ctctgcttcg ggctctggga gcccagaagg aagccatctc 440
Consensus TCTTCCCAGC CGTGGGAGCC CCTGCAGCTG CATGTGGATA AAGCCGTCAG TGGCCTTCGC AGCCTCACCA CTCTGCTTCG GGCTCTGGGA GCCCAGAAGG AAGCCATCTC
Conservation
Sequence3 ---------- ---------- ---------- ---------- ---------- ---------- ---------- ---------- ---------- ---------- ---------- 95
hEPO ccctccagat gcggcctcag ctgctccact ccgaacaatc actgctgaca ctttccgcaa actcttccga gtctactcca atttcctccg gggaaagctg aagctgtaca 550
Consensus CCCTCCAGAT GCGGCCTCAG CTGCTCCACT CCGAACAATC ACTGCTGACA CTTTCCGCAA ACTCTTCCGA GTCTACTCCA ATTTCCTCCG GGGAAAGCTG AAGCTGTACA
Conservation
Sequence3 ---------- ---------- ---------- -A 96
hEPO caggggaggc ctgcaggaca ggggacagat ga 582
Consensus CAGGGGAGGC CTGCAGGACA GGGGACAGAT GA
Conservation

# IM:1h

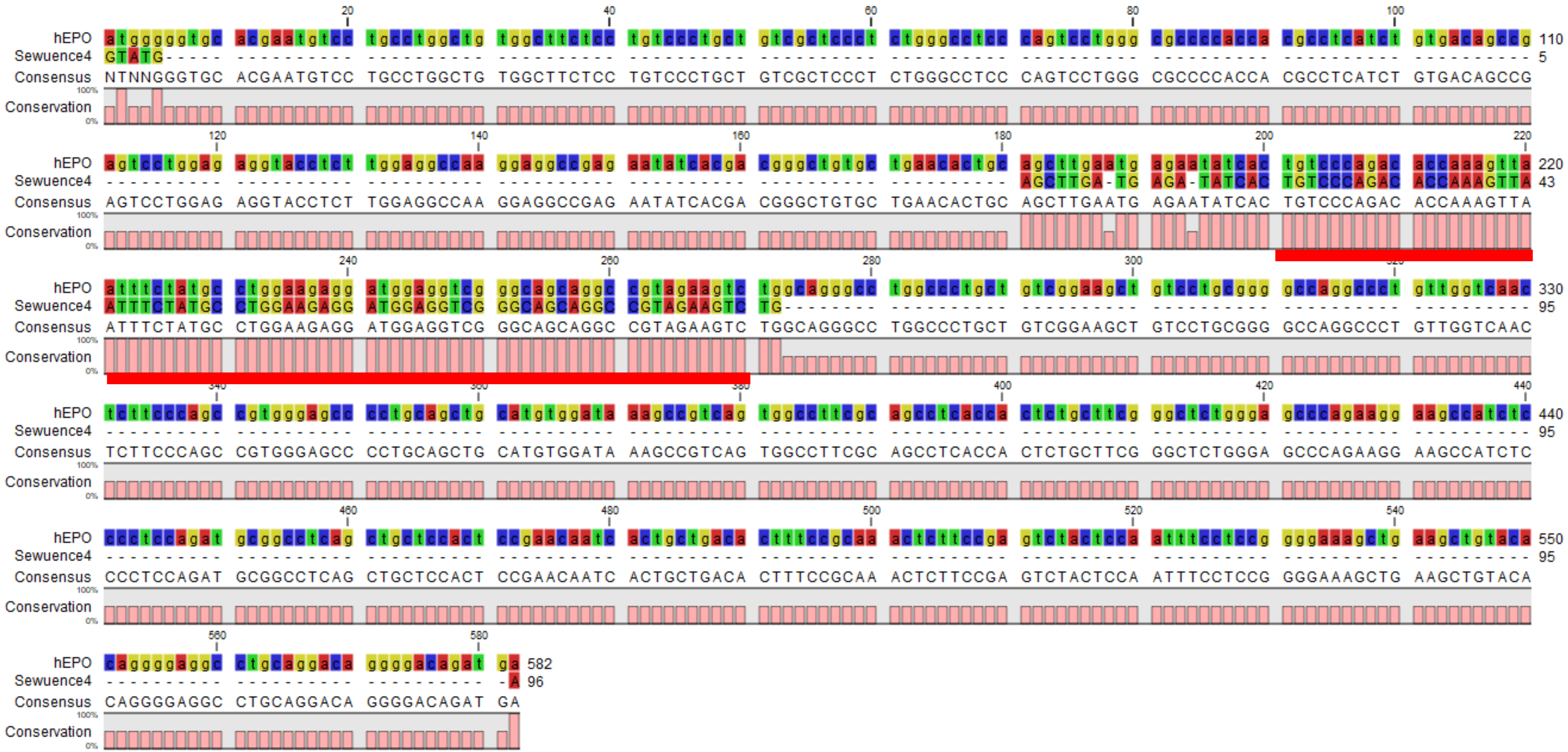

hEPO atgggggtgc acgaatgtcc tgcctggctg tggcttctcc tgtccctgct gtcgctccct ctgggcctcc cagtcctggg cgccccacca cgcctcatct gtgacagccg 110
Sewuence4 GTATG 5
Consensus NTNNGGGTGC ACGAATGTCC TGCCTGGCTG TGGCTTCTCC TGTCCCTGCT GTCGCTCCCT CTGGGCCTCC CAGTCCTGGG CGCCCCACCA CGCCTCATCT GTGACAGCCG
Conservation
hEPO agtcctggag aggtacctct tggaggccaa ggaggccgag aatatcacga cgggctgtgc tgaacactgc agcttgaatg agaatatcac tgtcccagac accaaagtta 220
Sewuence4 AGCTTGA-TG AGA-TATCAC TGTCCCAGAC ACCAAAGTTA 43
Consensus AGTCCTGGAG AGGTACCTCT TGGAGGCCAA GGAGGCCGAG AATATCACGA CGGGCTGTGC TGAACACTGC AGCTTGAATG AGAATATCAC TGTCCCAGAC ACCAAAGTTA
Conservation
hEPO atttctatgc ctggaagagg atggaggtcg ggcagcaggc cgtagaagtc tggcagggcc tggccctgct gtcggaagct gtcctgcggg gccaggccct gttggtcaac 330
Sewuence4 ATTTCTATGC CTGGAAGAGG ATGGAGGTCG GGCAGCAGGC CGTAGAAGTC TG 95
Consensus ATTTCTATGC CTGGAAGAGG ATGGAGGTCG GGCAGCAGGC CGTAGAAGTC TGGCAGGGCC TGGCCCTGCT GTCGGAAGCT GTCCTGCGGG GCCAGGCCCT GTTGGTCAAC
Conservation
hEPO tcttcccagc cgtgggagcc cctgcagctg catgtggata aagccgtcag tggccttcgc agcctcacca ctctgcttcg ggctctggga gcccagaagg aagccatctc 440
Sewuence4 95
Consensus TCTTCCCAGC CGTGGGAGCC CCTGCAGCTG CATGTGGATA AAGCCGTCAG TGGCCTTCGC AGCCTCACCA CTCTGCTTCG GGCTCTGGGA GCCCAGAAGG AAGCCATCTC
Conservation
hEPO ccctccagat gcggcctcag ctgctccact ccgaacaatc actgctgaca ctttccgcaa actcttccga gtctactcca atttcctccg gggaaagctg aagctgtaca 550
Sewuence4 95
Consensus CCCTCCAGAT GCGGCCTCAG CTGCTCCACT CCGAACAATC ACTGCTGACA CTTTCCGCAA ACTCTTCCGA GTCTACTCCA ATTTCCTCCG GGGAAAGCTG AAGCTGTACA
Conservation
hEPO caggggaggc ctgcaggaca ggggacagat ga 582
Sewuence4 -A 96
Consensus CAGGGGAGGC CTGCAGGACA GGGGACAGAT GA
Conservation

IM:2h

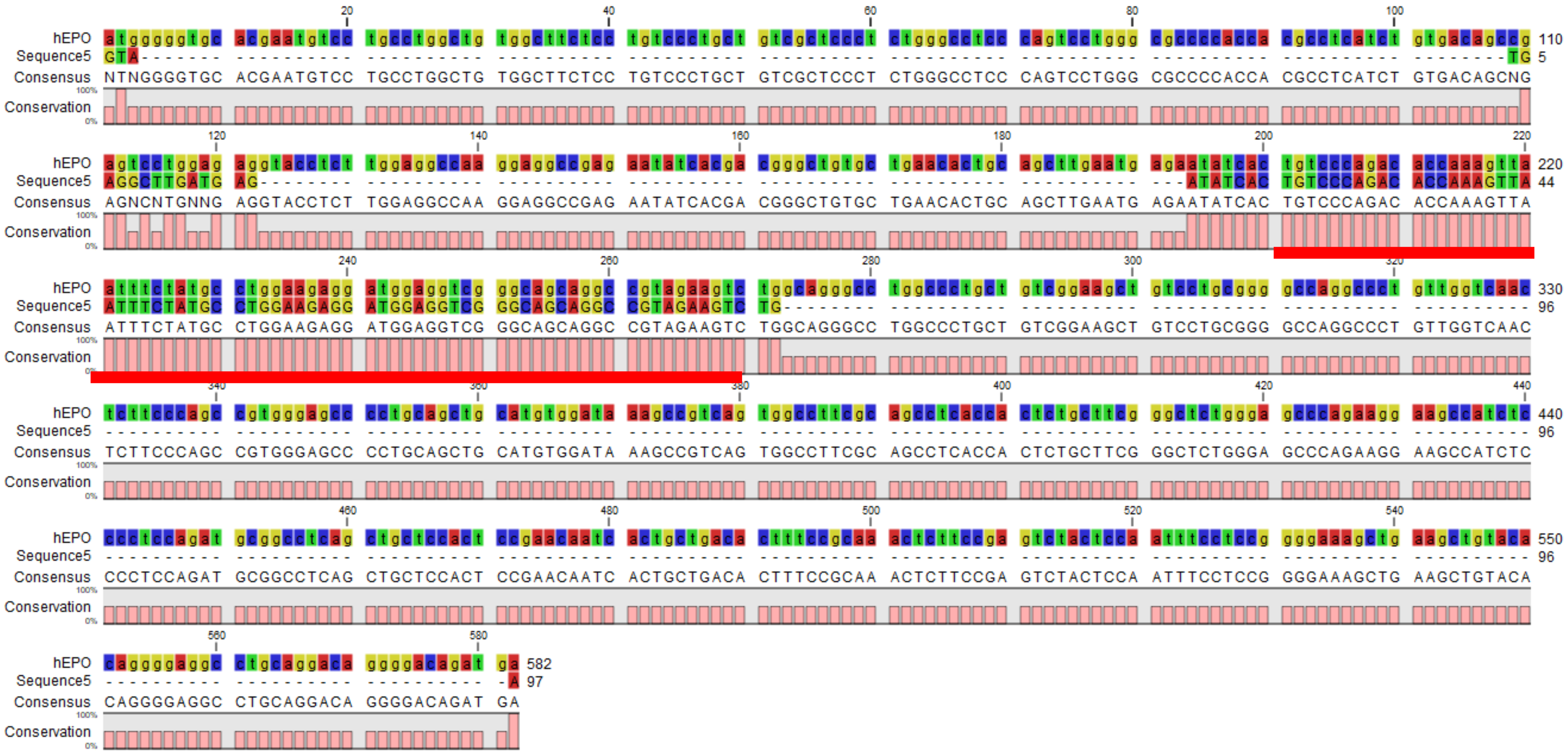
hEPO
Sequence5
Consensus
Conservation
NTNGGGGTGC ACGAATGTCC TGCCTGGCTG TGGCTTCTCC TGTCCCTGCT GTCGCTCCCT CTGGGCCTCC CAGTCCTGGG CGCCCCACCA CGCCTCATCT GTGACAGCNG
AGNCNTGNNG AGGTACCTCT TGGAGGCCAA GGAGGCCGAG AATATCACGA CGGGCTGTGC TGAACACTGC AGCTTGAATG AGAATATCAC TGTCCCAGAC ACCAAAGTTA
ATTTCTATGC CTGGAAGAGG ATGGAGGTCG GGCAGCAGGC CGTAGAAGTC TGGCAGGGCC TGGCCCTGCT GTCGGAAGCT GTCCTGCGGG GCCAGGCCCT GTTGGTCAAC
TCTTCCCAGC CGTGGGAGCC CCTGCAGCTG CATGTGGATA AAGCCGTCAG TGGCCTTCGC AGCCTCACCA CTCTGCTTCG GGCTCTGGGA GCCCAGAAGG AAGCCATCTC
CCCTCCAGAT GCGGCCTCAG CTGCTCCACT CCGAACAATC ACTGCTGACA CTTTCCGCAA ACTCTTCCGA GTCTACTCCA ATTTCCTCCG GGGAAAGCTG AAGCTGTACA
CAGGGGAGGC CTGCAGGACA GGGGACAGAT GA

IM:3h

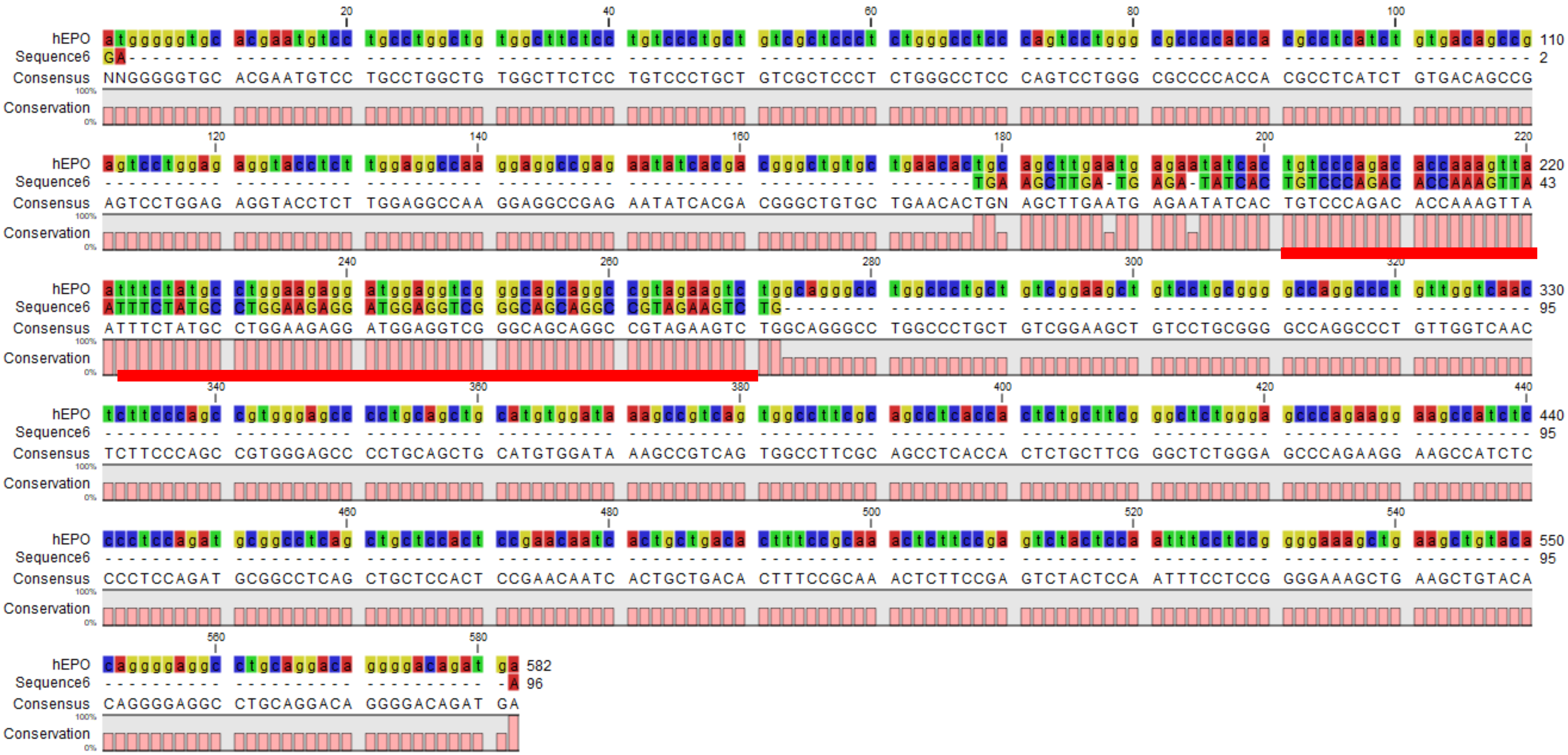
hEPO atgggggtgc acgaatgtcc tgcctggctg tggcttctcc tgtccctgct gtcgctccct ctgggcctcc cagtcctggg cgccccacca cgcctcatct gtgacagccg 110
Sequence6 GA-------- ---------- ---------- ---------- ---------- ---------- ---------- ---------- ---------- ---------- ---------- 2
Consensus NNGGGGTGC ACGAATGTCC TGCCTGGCTG TGGCTTCTCC TGTCCCTGCT GTCGCTCCCT CTGGGCCTCC CAGTCCTGGG CGCCCCACCA CGCCTCATCT GTGACAGCCG
Conservation
hEPO agtcctggag aggtacctct tggaggccaa ggaggccgag aatatcacga cgggctgtgc tgaacactgc agcttgaatg agaatatcac tgtcccagac accaaagtta 220
Sequence6 ---------- ---------- ---------- ---------- ---------- ---------- -------TGA AGCTTGA-TG AGA-TATCAC TGTCCCAGAC ACCAAAGTTA 43
Consensus AGTCCTGGAG AGGTACCTCT TGGAGGCCAA GGAGGCCGAG AATATCACGA CGGGCTGTGC TGAACACTGN AGCTTGAATG AGAATATCAC TGTCCCAGAC ACCAAAGTTA
Conservation
hEPO atttctatgc ctggaagagg atggaggtcg ggcagcaggc cgtagaagtc tggcagggcc tggccctgct gtcggaagct gtcctgcggg gccaggccct gttggtcaac 330
Sequence6 ATTTCTATGC CTGGAAGAGG ATGGAGGTCG GGCAGCAGGC CGTAGAAGTC TG-------- ---------- ---------- ---------- ---------- ---------- 95
Consensus ATTTCTATGC CTGGAAGAGG ATGGAGGTCG GGCAGCAGGC CGTAGAAGTC TGGCAGGGCC TGGCCCTGCT GTCGGAAGCT GTCCTGCGGG GCCAGGCCCT GTTGGTCAAC
Conservation
hEPO tcttcccagc cgtgggagcc cctgcagctg catgtggata aagccgtcag tggccttcgc agcctcacca ctctgcttcg ggctctggga gcccagaagg aagccatctc 440
Sequence6 ---------- ---------- ---------- ---------- ---------- ---------- ---------- ---------- ---------- ---------- ---------- 95
Consensus TCTTCCCAGC CGTGGGAGCC CCTGCAGCTG CATGTGGATA AAGCCGTCAG TGGCCTTCGC AGCCTCACCA CTCTGCTTCG GGCTCTGGGA GCCCAGAAGG AAGCCATCTC
Conservation
hEPO ccctccagat gcggcctcag ctgctccact ccgaacaatc actgctgaca ctttccgcaa actcttccga gtctactcca atttcctccg gggaaagctg aagctgtaca 550
Sequence6 ---------- ---------- ---------- ---------- ---------- ---------- ---------- ---------- ---------- ---------- ---------- 95
Consensus CCCTCCAGAT GCGGCCTCAG CTGCTCCACT CCGAACAATC ACTGCTGACA CTTTCCGCAA ACTCTTCCGA GTCTACTCCA ATTTCCTCCG GGGAAAGCTG AAGCTGTACA
Conservation
hEPO caggggaggc ctgcaggaca ggggacagat ga 582
Sequence6 ---------- ---------- ---------- -A 96
Consensus CAGGGGAGGC CTGCAGGACA GGGGACAGAT GA
Conservation

IP:3h

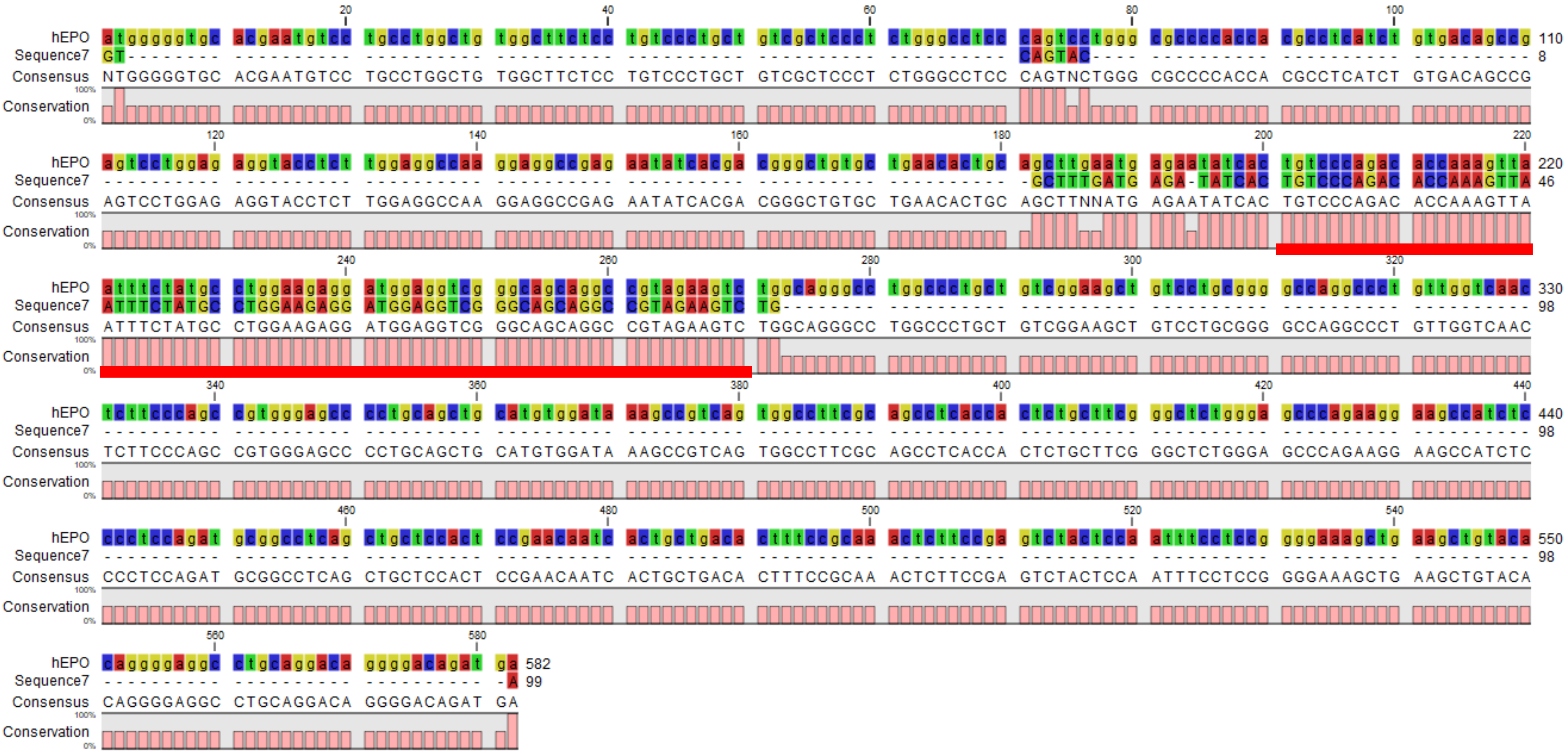

IP:2h

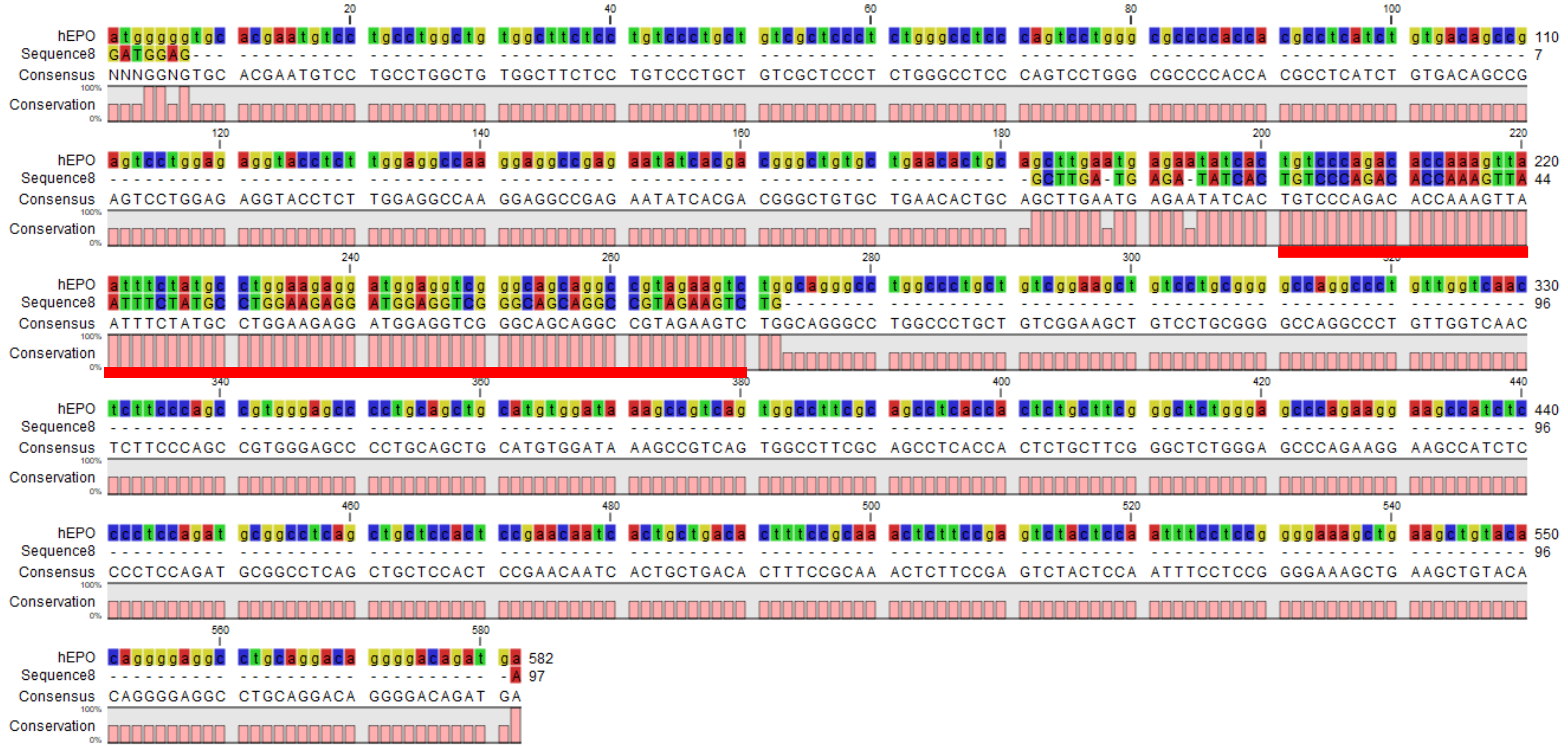
hEPO atggggtgc acgaatgtcc tgcctggctg tggcttctcc tgtccctgct gtcgctccct ctgggcctcc cagtcctggg cgccccacca cgcctcatct gtgacagccg 110
Sequence8 GATGGAG--- 7
Consensus NNNGGNGTGC ACGAATGTCC TGCCTGGCTG TGGCTTCTCC TGTCCCTGCT GTCGCTCCCT CTGGGCCTCC CAGTCCTGGG CGCCCCACCA CGCCTCATCT GTGACAGCCG
Conservation
hEPO agtcctggag aggtacctct tggaggccaa ggaggccgag aatatcacga cgggctgtgc tgaacactgc agcttgaatg agaatatcac tgtcccagac accaaagtta 220
Sequence8 -GCTTGA-TG AGA-TATCAC TGTCCCAGAC ACCAAAGTTA 44
Consensus AGTCCTGGAG AGGTACCTCT TGGAGGCCAA GGAGGCCGAG AATATCACGA CGGGCTGTGC TGAACACTGC AGCTTGAATG AGAATATCAC TGTCCCAGAC ACCAAAGTTA
Conservation
hEPO atttctatgc ctggaagagg atggaggtcg ggcagcaggc cgtagaagtc tggcagggcc tggccctgct gtcggaagct gtcctgcggg gccaggccct gttggtcaac 330
Sequence8 ATTTCTATGC CTGGAAGAGG ATGGAGGTCG GGCAGCAGGC CGTAGAAGTC TG 96
Consensus ATTTCTATGC CTGGAAGAGG ATGGAGGTCG GGCAGCAGGC CGTAGAAGTC TGGCAGGGCC TGGCCCTGCT GTCGGAAGCT GTCCTGCGGG GCCAGGCCCT GTTGGTCAAC
Conservation
hEPO tcttcccagc cgtgggagcc cctgcagctg catgtggata aagccgtcag tggccttcgc agcctcacca ctctgcttcg ggctctggga gcccagaagg aagccatctc 440
Sequence8 96
Consensus TCTTCCCAGC CGTGGGAGCC CCTGCAGCTG CATGTGGATA AAGCCGTCAG TGGCCTTCGC AGCCTCACCA CTCTGCTTCG GGCTCTGGGA GCCCAGAAGG AAGCCATCTC
Conservation
hEPO ccctccagat gcggcctcag ctgctccact ccgaacaatc actgctgaca ctttccgcaa actcttccga gtctactcca atttcctccg gggaaagctg aagctgtaca 550
Sequence8 96
Consensus CCCTCCAGAT GCGGCCTCAG CTGCTCCACT CCGAACAATC ACTGCTGACA CTTTCCGCAA ACTCTTCCGA GTCTACTCCA ATTTCCTCCG GGGAAAGCTG AAGCTGTACA
Conservation
hEPO caggggaggc ctgcaggaca ggggacagat ga 582
Sequence8 -A 97
Consensus CAGGGGAGGC CTGCAGGACA GGGGACAGAT GA
Conservation

# Stool

IV:1h

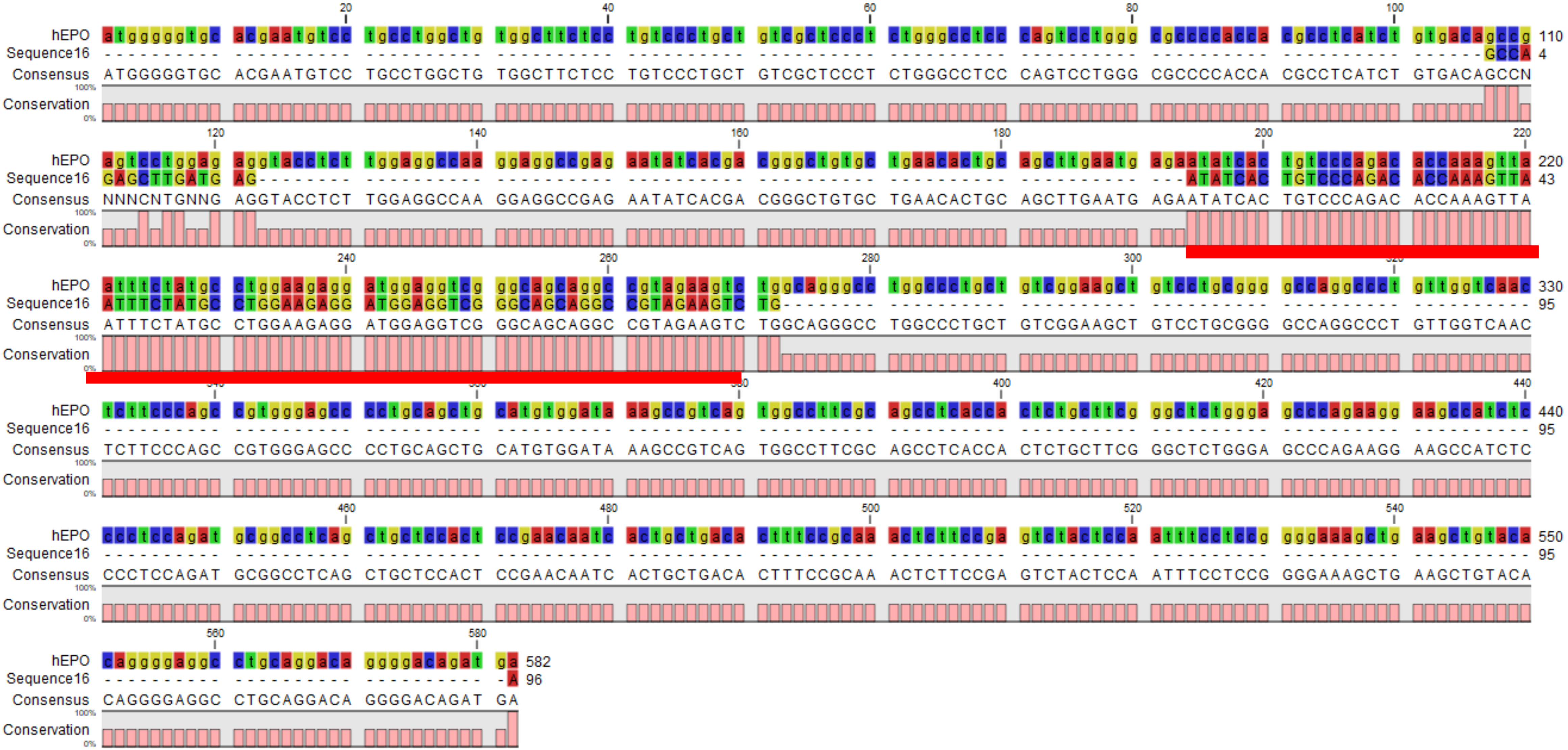
hEPO 110
Sequence16 GCCA 4
Consensus ATGGGGGTGC ACGAATGTCC TGCCTGGCTG TGGCTTCTCC TGTCCCTGCT GTCGCTCCCT CTGGGCCTCC CAGTCCTGGG CGCCCCACCA CGCCTCATCT GTGACAGCCN
Conservation
hEPO 220
Sequence16 GAGCTTGATG AG ATATCAC TGTCCCAGAC ACCAAAGTTA 43
Consensus NNNCNTGNNG AGGTACCTCT TGGAGGCCAA GGAGGCCGAG AATATCACGA CGGGCTGTGC TGAACACTGC AGCTTGAATG AGAATATCAC TGTCCCAGAC ACCAAAGTTA
Conservation
hEPO 330
Sequence16 ATTTCTATGC CTGGAAGAGG ATGGAGGTCG GGCAGCAGGC CGTAGAAGTC TG 95
Consensus ATTTCTATGC CTGGAAGAGG ATGGAGGTCG GGCAGCAGGC CGTAGAAGTC TGGCAGGGCC TGGCCCTGCT GTCGGAAGCT GTCCTGCGGG GCCAGGCCCT GTTGGTCAAC
Conservation
hEPO 440
Sequence16 95
Consensus TCTTCCCAGC CGTGGGAGCC CCTGCAGCTG CATGTGGATA AAGCCGTCAG TGGCCTTCGC AGCCTCACCA CTCTGCTTCG GGCTCTGGGA GCCCAGAAGG AAGCCATCTC
Conservation
hEPO 550
Sequence16 95
Consensus CCCTCCAGAT GCGGCCTCAG CTGCTCCACT CCGAACAATC ACTGCTGACA CTTTCCGCAA ACTCTTCCGA GTCTACTCCA ATTTCCTCCG GGGAAAGCTG AAGCTGTACA
Conservation
hEPO 582
Sequence16 A 96
Consensus CAGGGGAGGC CTGCAGGACA GGGGACAGAT GA
Conservation

IV:2h

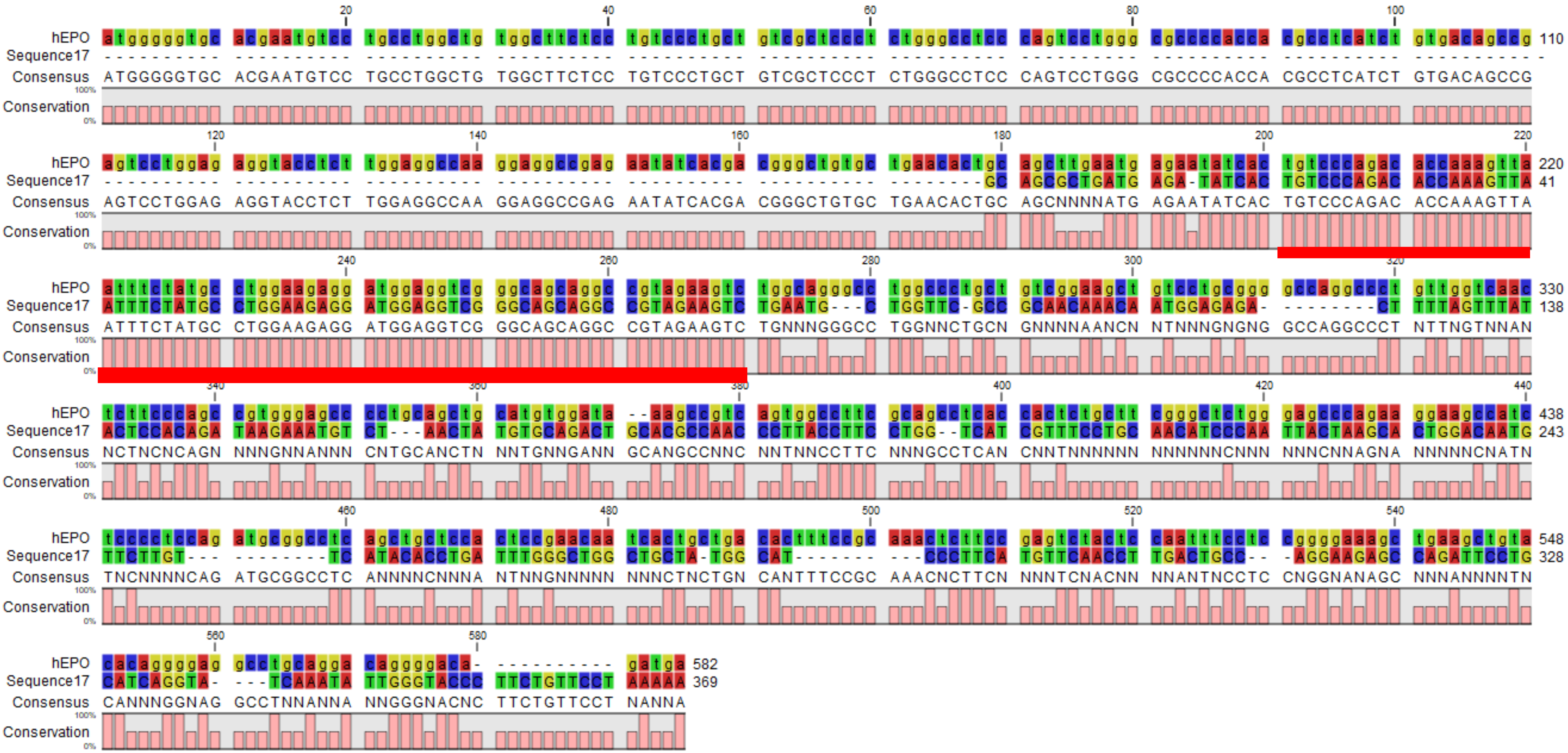
hEPO
Sequence17
Consensus
Conservation
100%
0%
20
40
60
80
100
110
120
140
160
180
200
220
220
41
240
260
280
300
320
330
138
340
360
380
400
420
440
438
243
460
480
500
520
540
548
328
560
580
582
369

IV:3h

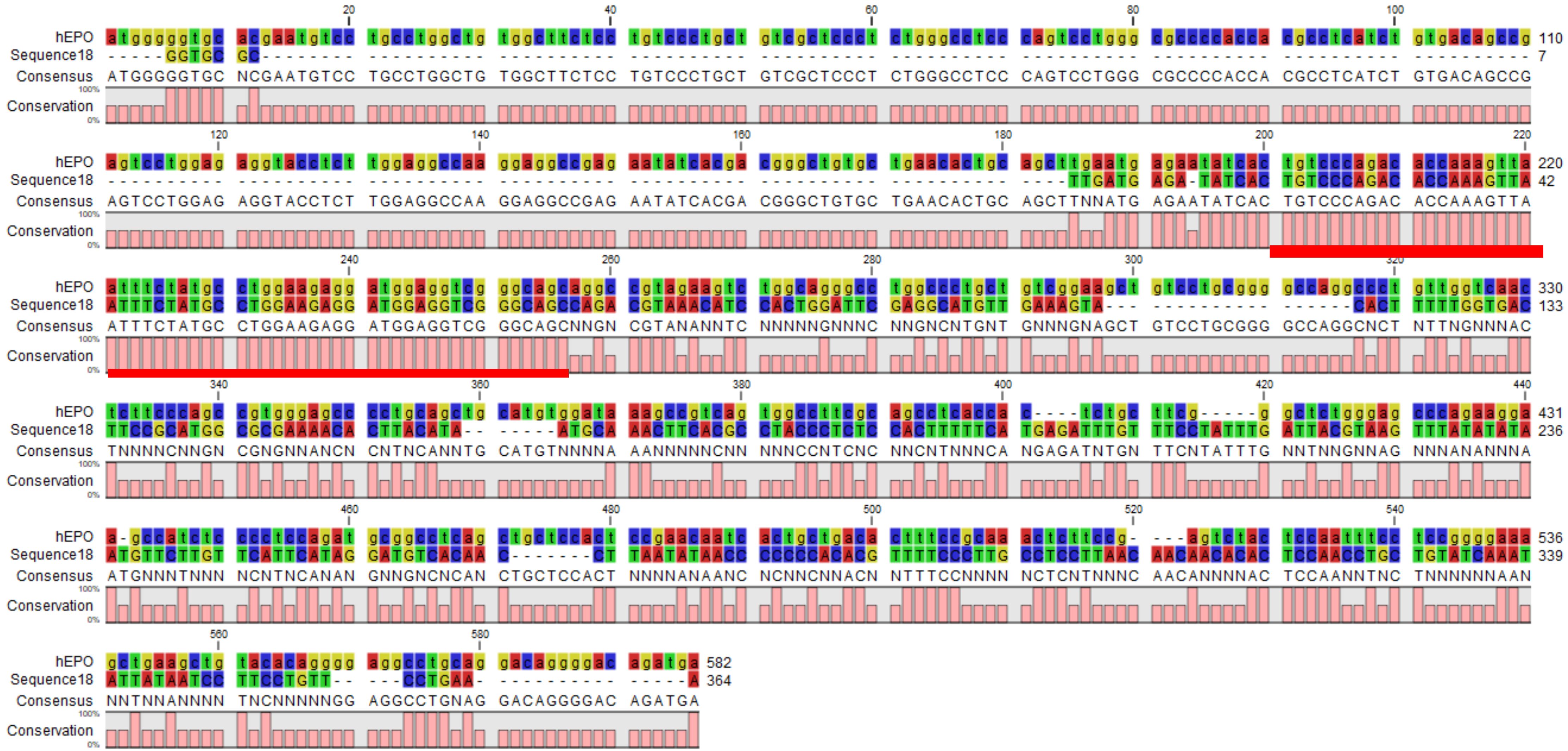

hEPO 110
Sequence18 7
Consensus ATGGGGTGC NCGAATGTCC TGCCTGGCTG TGGCTTCTCC TGTCCCTGCT GTCGCTCCCT CTGGGCCTCC CAGTCCTGGG CGCCCCACCA CGCCTCATCT GTGACAGCCG
Conservation
hEPO 220
Sequence18 42
Consensus AGTCCTGGAG AGGTACCTCT TGGAGGCCAA GGAGGCCGAG AATATCACGA CGGGCTGTGC TGAACACTGC AGCTTNNATG AGAATATCAC TGTCCCAGAC ACCAAAGTTA
Conservation
hEPO 330
Sequence18 133
Consensus ATTTCTATGC CTGGAAGAGG ATGGAGGTCG GGCAGCNNGN CGTANANNTC NNNNNGNNNC NNGNCNTGNT GNNNGNAGCT GTCCTGCGGG GCCAGGCNCT NTTNGNNNAC
Conservation
hEPO 431
Sequence18 236
Consensus TNNNNCNNGN CGNGNNANCN CNTNCANNTG CATGTNNNNA AANNNNNCNN NNNCCNTCNC NNCNTNNNCA NGAGATNTGN TTCNTATTTG NNTNNGNNAG NNNANANNNA
Conservation
hEPO 536
Sequence18 339
Consensus ATGNNNTNNN NCNTNCANAN GNNGNCNCAN CTGCTCCACT NNNNANAANC NCNNCNNACN NTTTCCNNNN NCTCNTNNNC AACANNNNAC TCCAANNTNC TNNNNNNAAN
Conservation
hEPO 582
Sequence18 364
Consensus NNTNNANNNN TNCNNNNNGG AGGCCTGNAG GACAGGGGAC AGATGA
Conservation

IM:1h

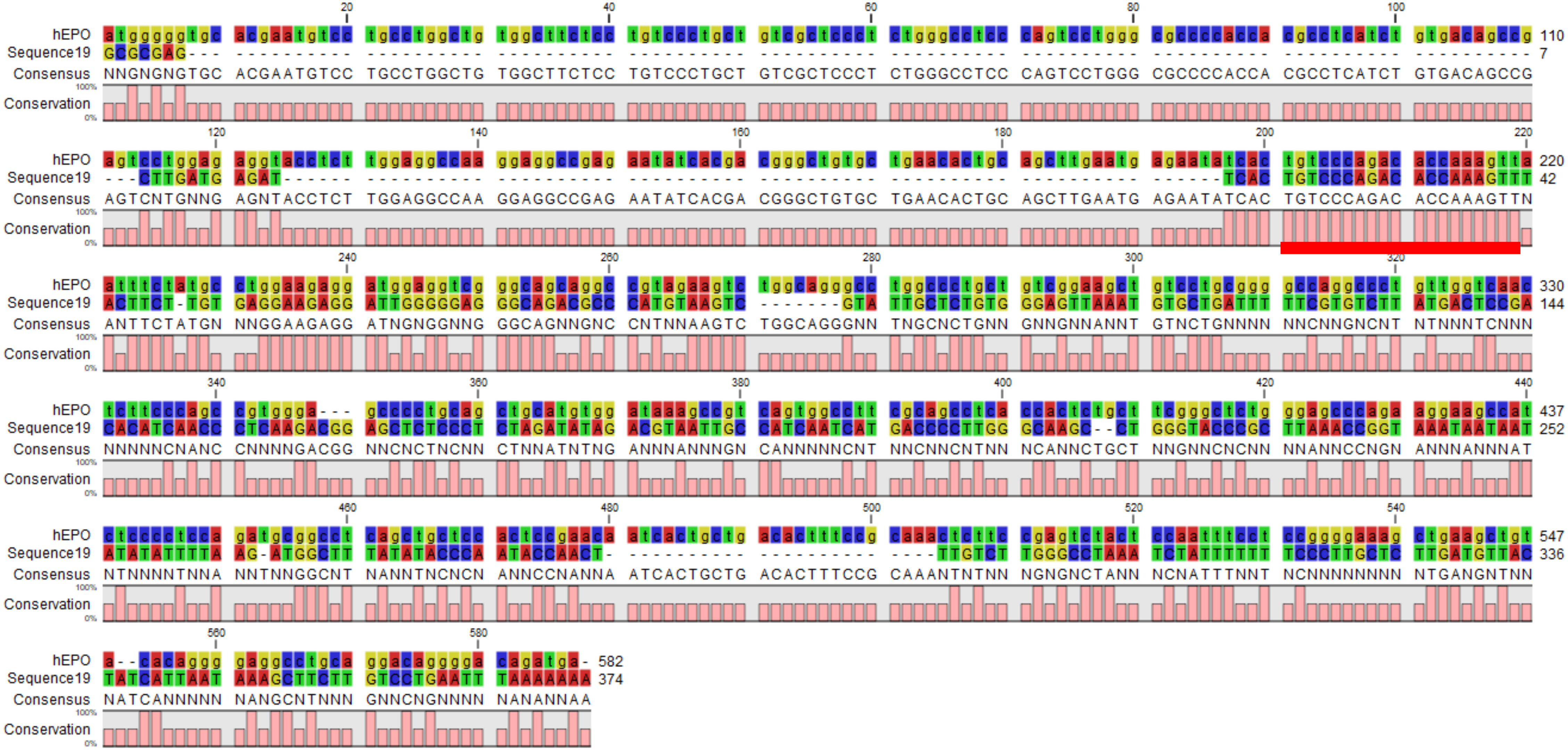
20 40 60 80 100
hEPO atgggggtgc acgaatgtcc tgcctggctg tggcttctcc tgtccctgct gtcgctccct ctgggcctcc cagtcctggg cgccccacca cgcctcatct gtgacagccg 110
Sequence19 GCGCGAG--- ---------- ---------- ---------- ---------- ---------- ---------- ---------- ---------- ---------- ---------- 7
Consensus NNGNGNGTGC ACGAATGTCC TGCCTGGCTG TGGCTTCTCC TGTCCCTGCT GTCGCTCCCT CTGGGCCTCC CAGTCCTGGG CGCCCCACCA CGCCTCATCT GTGACAGCCG
Conservation 100% 0%
120 140 160 180 200 220
hEPO agtcctggag aggtacctct tggaggccaa ggaggccgag aatatcacga cgggctgtgc tgaacactgc agcttgaatg agaatatcac tgtcccagac accaaagtta 220
Sequence19 ---CTTGATG AGAT------ ---------- ---------- ---------- ---------- ---------- ---------- ------TCAC TGTCCCAGAC ACCAAAGTTT 42
Consensus AGTCNTGNNG AGNTACCTCT TGGAGGCCAA GGAGGCCGAG AATATCACGA CGGGCTGTGC TGAACACTGC AGCTTGAATG AGAATATCAC TGTCCCAGAC ACCAAAGTTN
Conservation 100% 0%
240 260 280 300 320
hEPO atttctatgc ctggaagagg atggaggtcg ggcagcaggc cgtagaagtc tggcagggcc tggccctgct gtcggaagct gtcctgcggg gccaggccct gttggtcaac 330
Sequence19 ACTTCT-TGT GAGGAAGAGG ATTGGGGGAG GGCAGACGCC CATGTAAGTC -------GTA TTGCTCTGTG GGAGTTAAAT GTGCTGATTT TTCGTGTCTT ATGACTCCGA 144
Consensus ANTTCTATGN NNGGAAGAGG ATNGNGGNNG GGCAGNNGNC CNTNNAAGTC TGGCAGGGNN TNGCNCTGNN GNNGNNANNT GTNCTGNNNN NNCNNGNCNT NTNNNTCNNN
Conservation 100% 0%
340 360 380 400 420 440
hEPO tcttcccagc cgtggga--- gcccctgcag ctgcatgtgg ataaagccgt cagtggcctt cgcagcctca ccactctgct tcgggctctg ggagcccaga aggaagccat 437
Sequence19 CACATCAACC CTCAAGACGG AGCTCTCCCT CTAGATATAG ACGTAATTGC CATCAATCAT GACCCCTTGG GCAAGC--CT GGGTACCCGC TTAAACCGGT AAATAATAAT 252
Consensus NNNNNCNANC CNNNNGACGG NNCNCTNCNN CTNNATNTNG ANNNANNNGN CANNNNNCNT NNCNNCNTNN NCANNCTGCT NNGNNCNCNN NNANNCCNGN ANNNANNNAT
Conservation 100% 0%
460 480 500 520 540
hEPO ctcccctcca gatgcggcct cagctgctcc actccgaaca atcactgctg acactttccg caaactcttc cgagtctact ccaatttcct ccggggaaag ctgaagctgt 547
Sequence19 ATATATTTTA AG-ATGGCTT TATATACCCA ATACCAACT- ---------- ---------- ----TTGTCT TGGGCCTAAA TCTATTTTTT TCCCTTGCTC TTGATGTTAC 336
Consensus NTNNNNTNNA NNTNNGGCNT NANNTNCNCN ANNCCNANNA ATCACTGCTG ACACTTTCCG CAAANTNTNN NGNGNCTANN NCNATTTNNT NCNNNNNNNN NTGANGNTNN
Conservation 100% 0%
560 580
hEPO a--cacaggg gaggcctgca ggacagggga cagatga- 582
Sequence19 TATCATTAAT AAAGCTTCTT GTCCTGAATT TAAAAAAA 374
Consensus NATCANNNNN NANGCNTNNN GNNCNGNNNN NANANNAA
Conservation 100% 0%

IM:2h

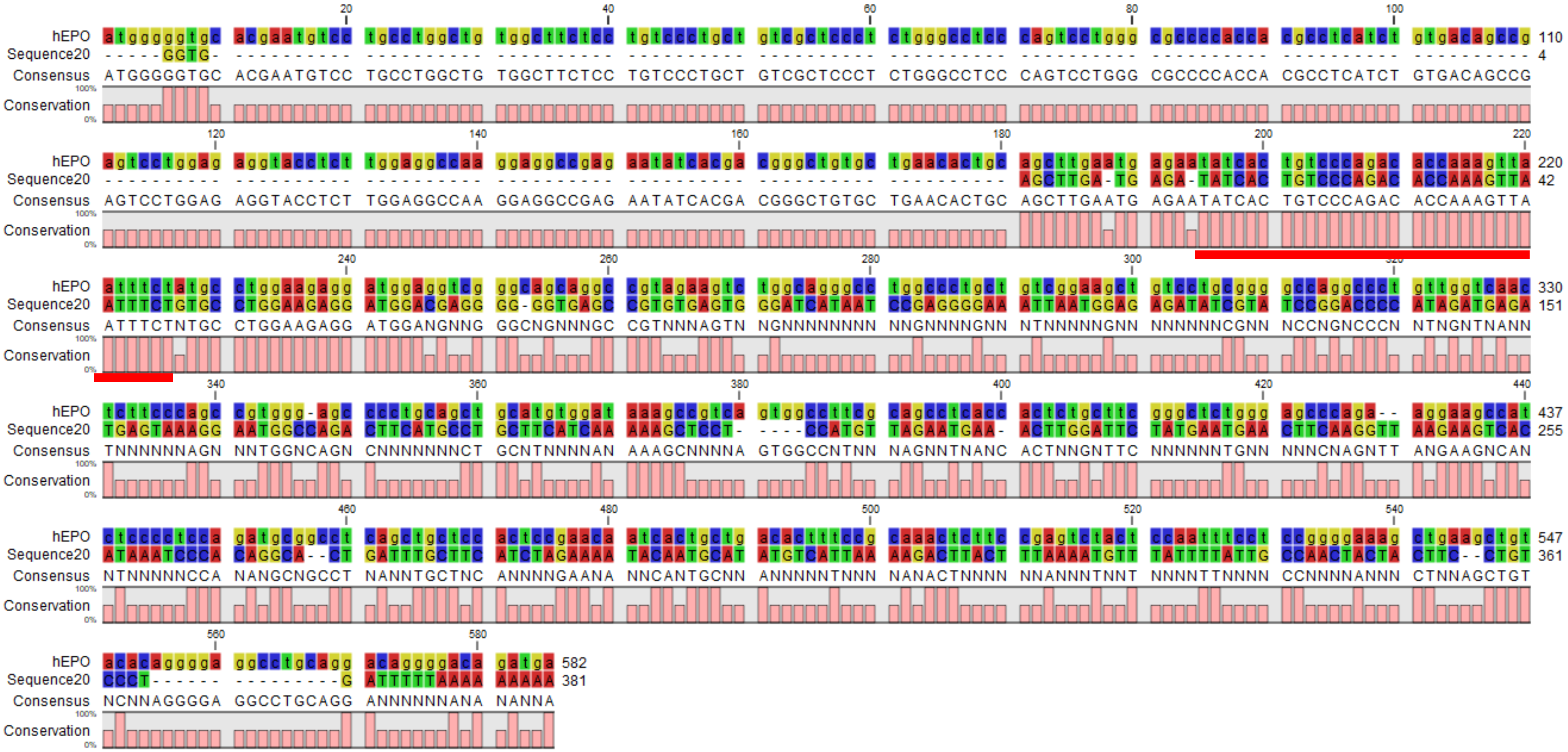

hEPO
Sequence20
Consensus
Conservation
100%
0%
ATGGGGTGC ACGAATGTCC TGCCTGGCTG TGGCTTCTCC TGTCCCTGCT GTCGCTCCCT CTGGGCCTCC CAGTCCTGGG CGCCCCACCA CGCCTCATCT GTGACAGCCG
AGTCCTGGAG AGGTACCTCT TGGAGGCCAA GGAGGCCGAG AATATCACGA CGGGCTGTGC TGAACACTGC AGCTTGAATG AGAATATCAC TGTCCCAGAC ACCAAAGTTA
ATTTCTNTGC CTGGAAGAGG ATGGANGNNG GGCNGNNNGC CGTNNNAGTN NGNNNNNNNN NNGNNNNGNN NTNNNNNGNN NNNNNNCGNN NCCNGNCCCN NTNGNTNANN
TNNNNNNAGN NNTGGNCAGN CNNNNNNNCT GCNTNNNNAN AAAGCNNNNA GTGGCCNTNN NAGNNTNANC ACTNNGNTTC NNNNNNTGNN NNNCNAGNTT ANGAAGNCAN
NTNNNNNCCA NANGCNGCCT NANNTGCTNC ANNNNGAANA NNCANTGCNN ANNNNNTNNN NANACTNNNN NNANNNTNNT NNNNTTNNNN CCNNNNANNN CTNNAGCTGT
NCNNAGGGGA GGCCTGCAGG ANNNNNNANA NANNA

IM:3h

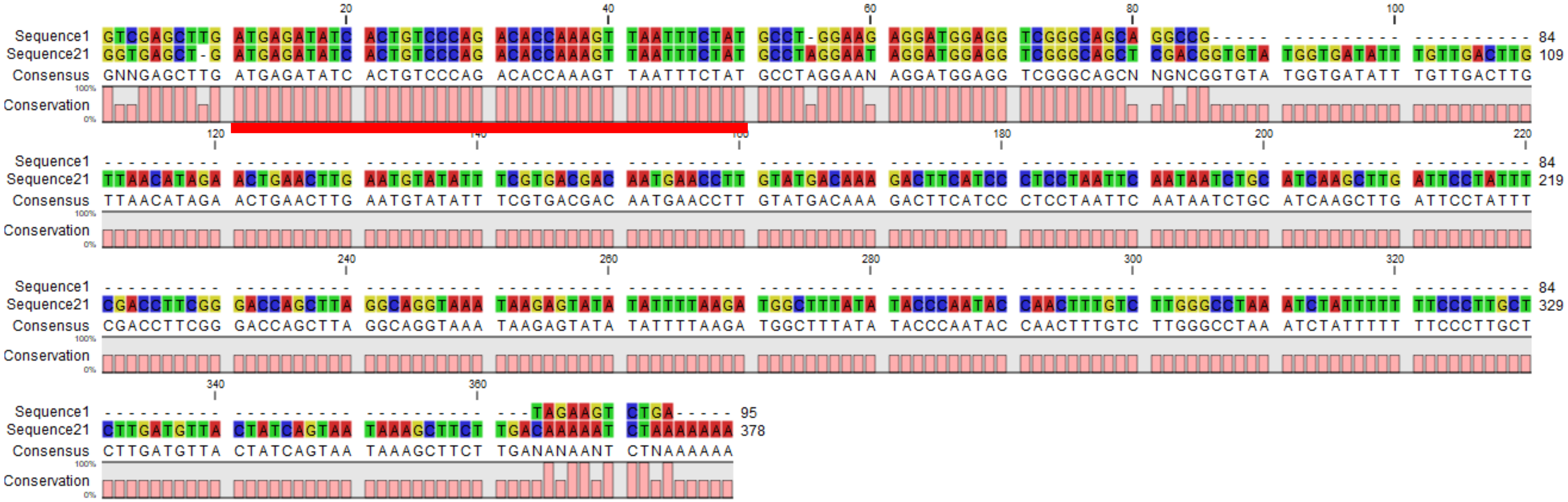

IP:1h

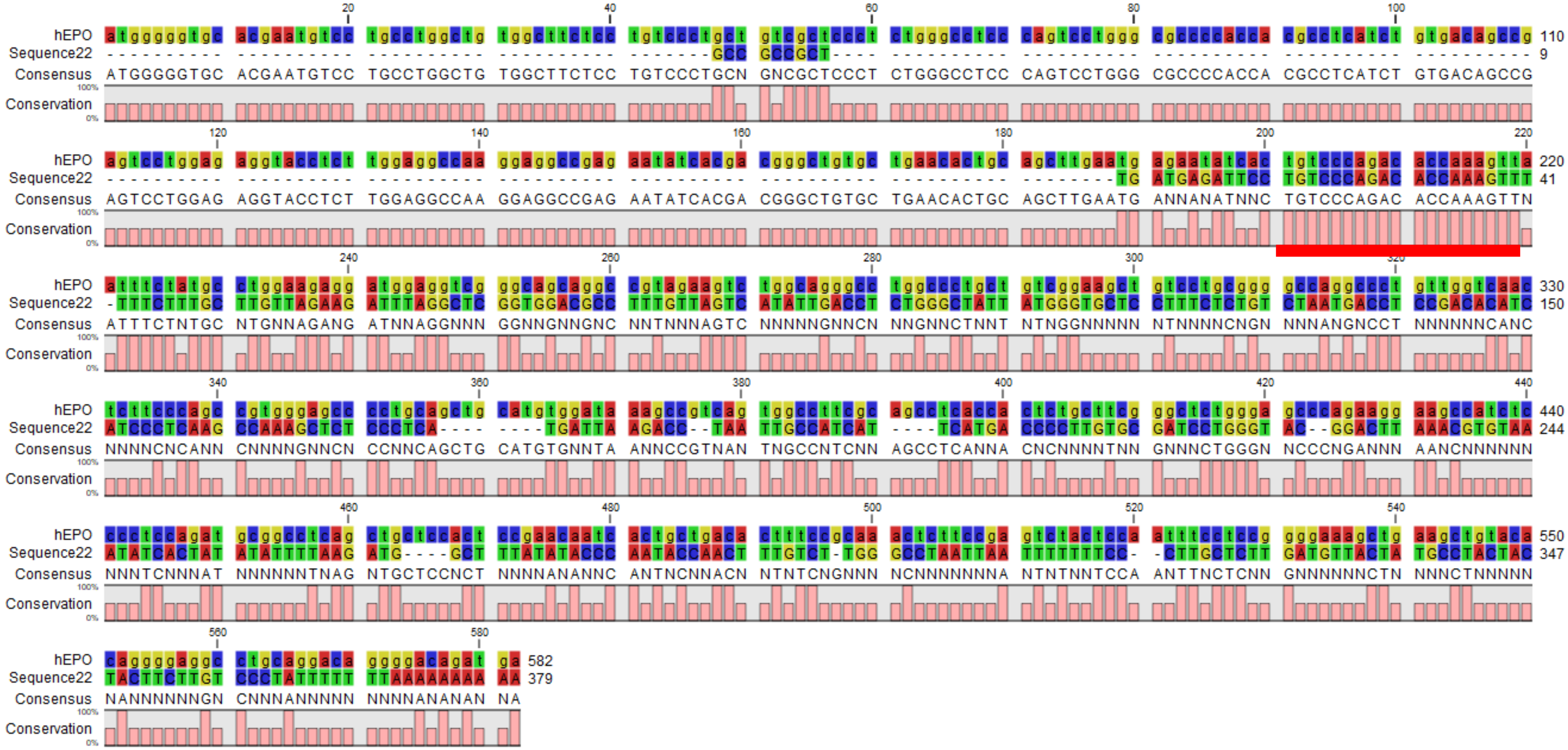
hEPO
Sequence22
Consensus
Conservation
ATGGGGTGC ACGAATGTCC TGCCTGGCTG TGGCTTCTCC TGTCCCTGCN GNCGCTCCCT CTGGGCCTCC CAGTCCTGGG CGCCCCACCA CGCCTCATCT GTGACAGCCG
AGTCCTGGAG AGGTACCTCT TGGAGGCCAA GGAGGCCGAG AATATCACGA CGGGCTGTGC TGAACACTGC AGCTTGAATG ANNANATNNC TGTCCCAGAC ACCAAAGTTN
ATTTCTNTGC NTGNNAGANG ATNNAGGNNN GGNNGNNGNC NNTNNNAGTC NNNNNGNNCN NNGNNCTNNT NTNGGNNNNN NTNNNNCNGN NNNANGNCCT NNNNNNCANC
NNNNCNCANN CNNNNGNNCN CCNNCAGCTG CATGTGNNTA ANNCCGTNAN TNGCCNTCNN AGCCTCANNA CNCNNNNTNN GNNNCTGGGN NCCCNGANNN AANCNNNNNN
NNNTCNNNAT NNNNNNTNAG NTGCTCCNCT NNNNANANNC ANTNCNNACN NTNTCNGNNN NCNNNNNNNA NTNTNNTCCA ANTTNCTCNN GNNNNNCTN NNNCTNNNNN
NANNNNNNGN CNNNANNNNN NNNNANANAN NA

IP:2h

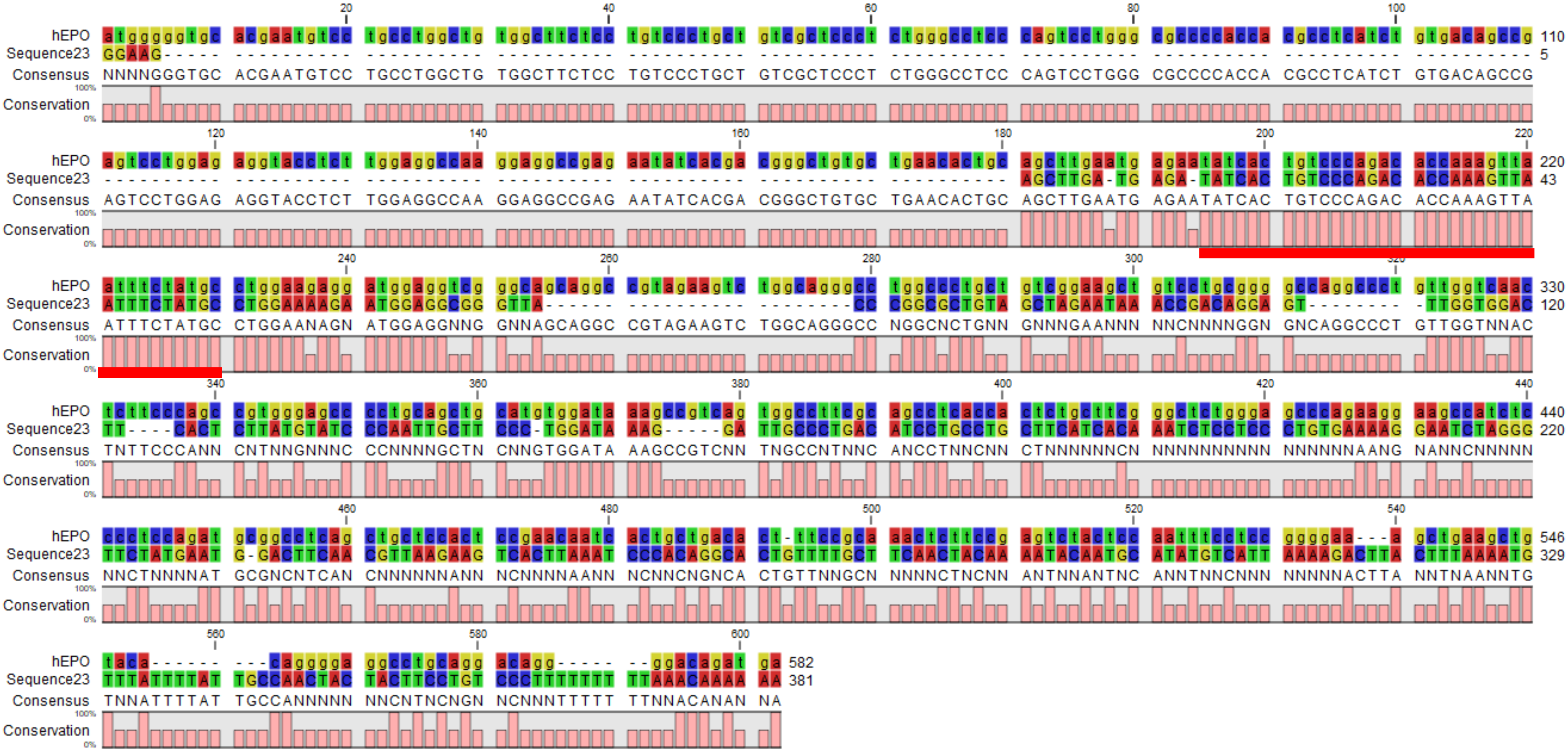
hEPO
Sequence23
Consensus
Conservation
NNNNGGGTGC ACGAATGTCC TGCCTGGCTG TGGCTTCTCC TGTCCCTGCT GTCGCTCCCT CTGGGCCTCC CAGTCCTGGG CGCCCCACCA CGCCTCATCT GTGACAGCCG
AGTCCTGGAG AGGTACCTCT TGGAGGCCAA GGAGGCCGAG AATATCACGA CGGGCTGTGC TGAACACTGC AGCTTGAATG AGAATATCAC TGTCCCAGAC ACCAAAGTTA
ATTTCTATGC CTGGAANAGN ATGGAGGNNG GNNAGCAGGC CGTAGAAGTC TGGCAGGGCC NGGCNCTGNN GNNNGAANNN NNCNNNNGGN GNCAGGCCCT GTTGGTNNAC
TNTTCCCANN CNTNNGNNNC CCNNNNGCTN CNNGTGGATA AAGCCGTCNN TNGCCNTNNC ANCCTNNCNN CTNNNNNNCN NNNNNNNNNN NNNNNNAANG NANNCNNNNN
NNCTNNNNAT GCGNCNTCAN CNNNNNNANN NCNNNNAANN NCNNCNGNCA CTGTTNNGCN NNNNCTNCNN ANTNNANTNC ANNTNNCNNN NNNNNACTTA NNTNAANNTG
TNNATTTTAT TGCCANNNNN NNCNTNCNGN NCNNNTTTTT TTNNACANAN NA

IP:3h

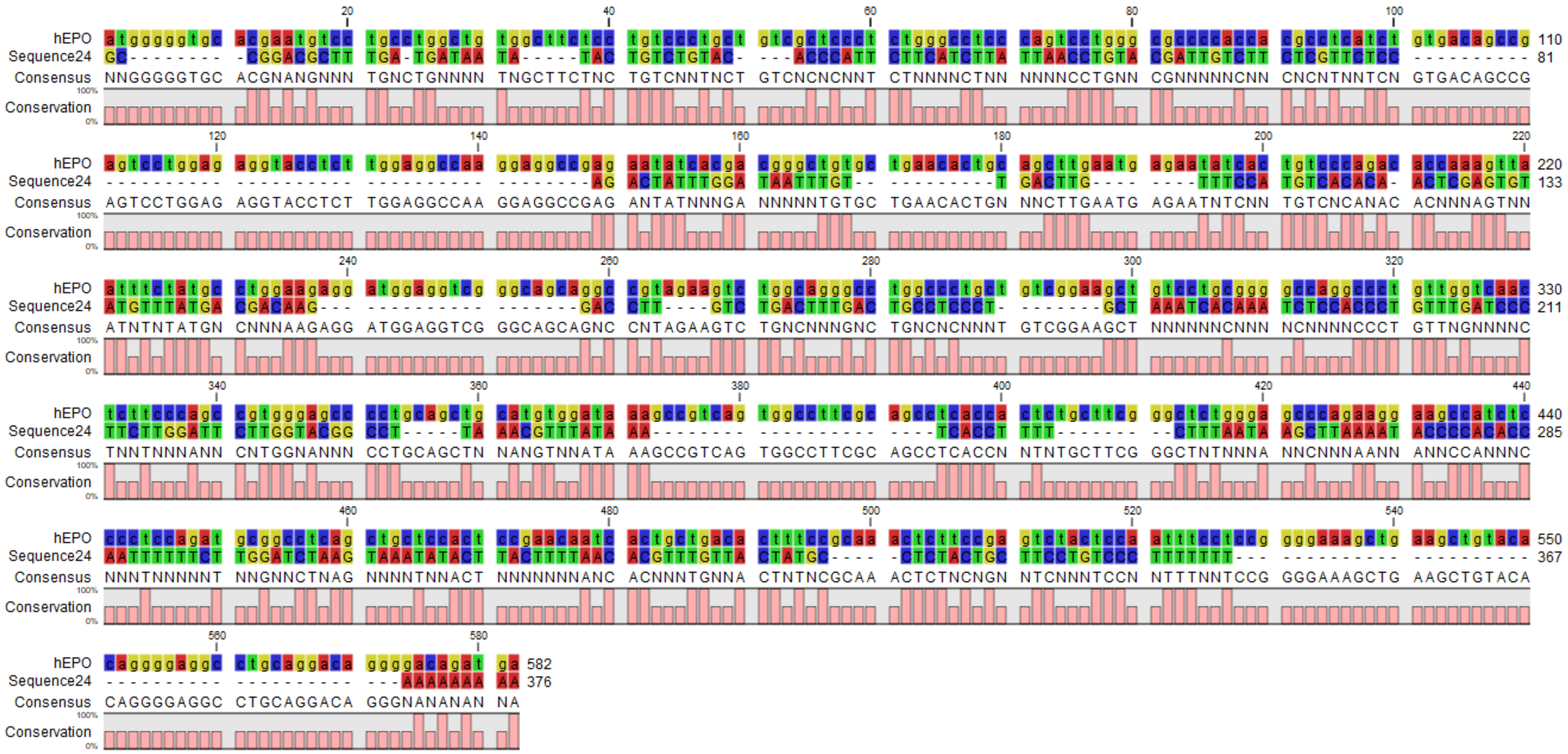

Supplement: Supplemental Information 1 [file peerj-08-8595-s001.pdf]
