# Supplementary figures and images for: The detection of trans gene fragments of hEPO in gene doping model mice by Taqman qPCR assay
Source: PeerJ. 2020 Feb 25;8:e8595. doi: 10.7717/peerj.8595 (PMC7047860; doi:10.7717/peerj.8595)

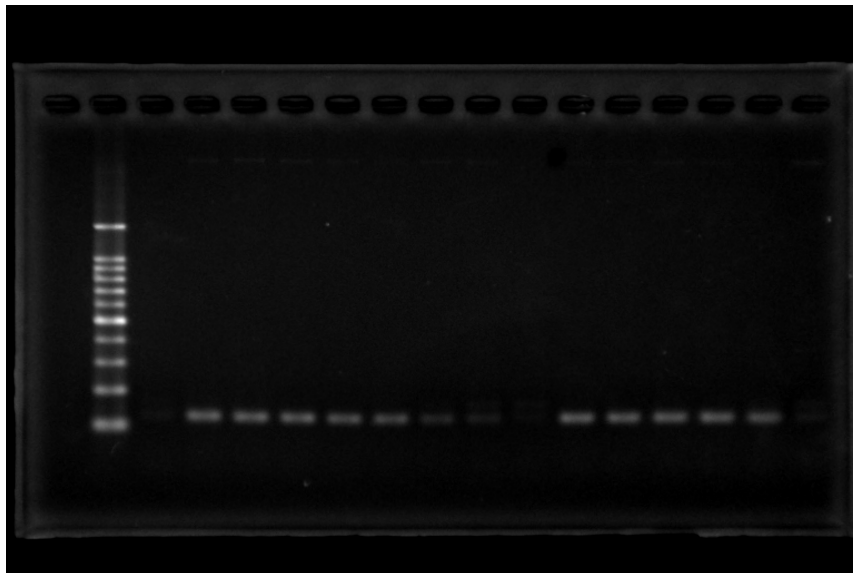

Supplement: Supplemental Information 3 — A single band was detected from IV and IM samples. [file peerj-08-8595-s003.pdf]

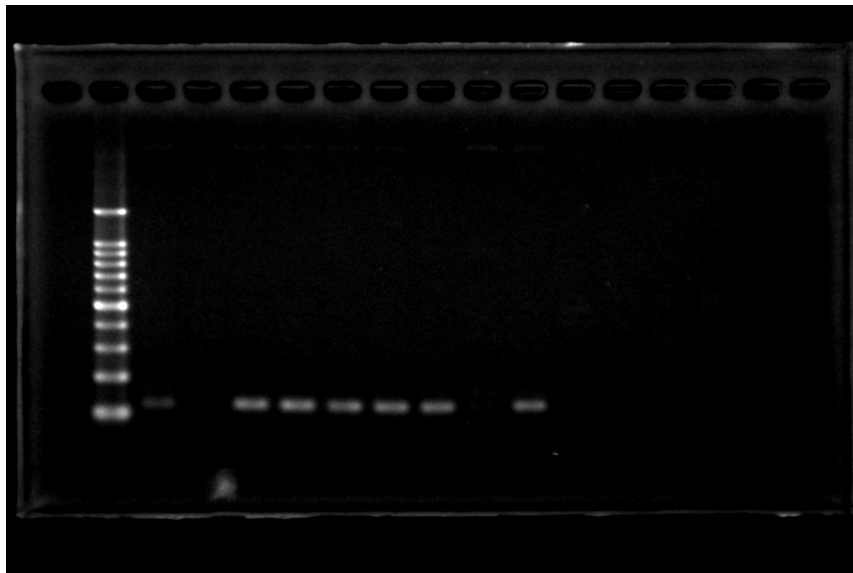

Supplement: Supplemental Information 4 — A single band was detected from blood samples. There was no band in the negative control (human cells). [file peerj-08-8595-s004.pdf]

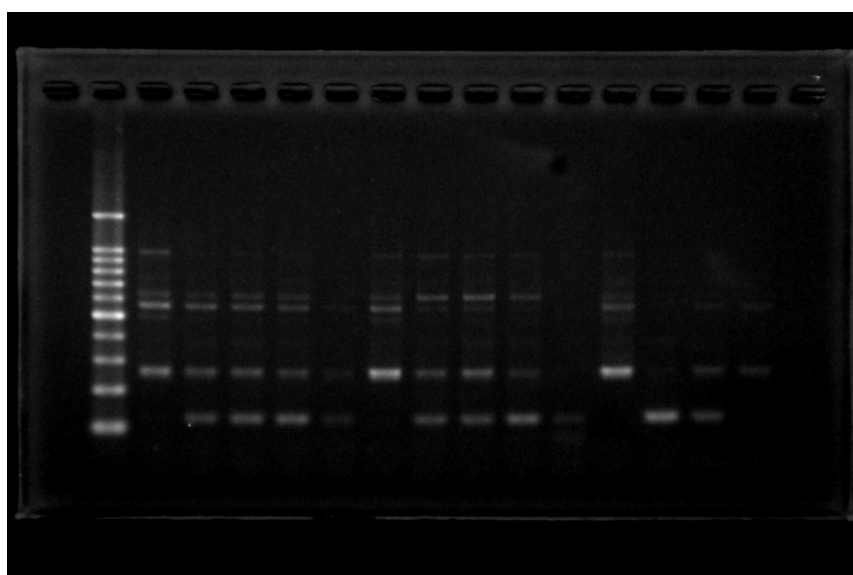

Supplement: Supplemental Information 5 — Some bands were detected from stool samples. [file peerj-08-8595-s005.pdf]
